# Establishment of novel long-term cultures from EpCAM positive and negative circulating tumour cells from patients with metastatic gastroesophageal cancer

**DOI:** 10.1038/s41598-019-57164-6

**Published:** 2020-01-17

**Authors:** Daniel Brungs, Elahe Minaei, Ann-Katrin Piper, Jay Perry, Ashleigh Splitt, Martin Carolan, Shantay Ryan, Xiao Juan Wu, Stéphanie Corde, Moeava Tehei, Morteza Aghmesheh, Kara L. Vine, Therese M. Becker, Marie Ranson

**Affiliations:** 1 Illawarra Health and Medical Research Institute, Wollongong, Australia; 2 School of Chemistry and Molecular Bioscience, University of Wollongong, Wollongong, Australia; 3 Illawarra Cancer Centre, Wollongong Hospital, Wollongong, Australia; 4 CONCERT-Translational Cancer Research Centre, New South Wales, Australia; 5 NSWHP Anatomical Pathology Liverpool Hospital, Sydney, Australia; 6 School of Medicine, University of Western Sydney, Sydney, Australia; 7 Centre for Circulating Tumour Cell Diagnostics and Research, Ingham Institute for Applied Medical Research, Sydney, Australia; 8 Radiation Oncology Department, Prince of Wales Hospital, Sydney, Australia; 9 Centre for Medical Radiation Physics, University of Wollongong, Wollongong, NSW Australia

**Keywords:** Cancer, Molecular biology, Oncology

## Abstract

Circulating tumour cell (CTC) enumeration and profiling has been established as a valuable clinical tool in many solid malignancies. A key challenge in CTC research is the limited number of cells available for study. *Ex vivo* CTC culture permits expansion of these rare cell populations for detailed characterisation, functional assays including drug sensitivity testing, and investigation of the pathobiology of metastases. We report for the first time the establishment and characterisation of two continuous CTC lines from patients with gastroesophageal cancer. The two cell lines (designated UWG01CTC and UWG02CTC) demonstrated rapid tumorigenic growth in immunodeficient mice and exhibit distinct genotypic and phenotypic profiles which are consistent with the tumours of origin. UWG02CTC exhibits an EpCAM+, cytokeratin+, CD44+ phenotype, while UWG01CTC, which was derived from a patient with metastatic neuroendocrine cancer, displays an EpCAM−, weak cytokeratin phenotype, with strong expression of neuroendocrine markers. Further, the two cell lines show distinct differences in drug and radiation sensitivity which match differential cancer-associated gene expression pathways. This is strong evidence implicating EpCAM negative CTCs in metastasis. These novel, well characterised, long-term CTC cell lines from gastroesophageal cancer will facilitate ongoing research into metastasis and the discovery of therapeutic targets.

## Introduction

Gastroesophageal cancers are among the most common and lethal cancers worldwide^1^. Most patients present with locally advanced or metastatic disease, or develop recurrent disease following curative surgery^2^. While many systemic treatment options are available, the prognosis of advanced gastroesophageal cancer remains poor, with median survival of less than 1 year^3^. Greater than 90% of gastric and gastroesophageal junction cancers are adenocarcinomas, with gastrointestinal stroma tumours (GIST), lymphomas, and neuroendocrine tumours found in a small minority of cases^4^.

Most patients with gastroesophageal cancer will require systemic treatment at some point in their treatment^3^. There is an increasing recognition of the limitations of using primary tumour features to guide systemic cancer treatment, due to tumour heterogeneity and the frequent disparity observed between primary and metastatic sites^5^. Metastatic biopsies are rarely undertaken however, due to both inaccessibility of metastatic sites and procedure morbidity. Circulating tumour cells (CTCs) are the likely intermediates of metastasis dissemination of cancer, and as such, can be expected to include the subpopulations which are responsible for disease progression^6^. While CTC enumeration has an established prognostic role, the true promise of CTCs is to provide a ‘real time’ view of the cancer using only peripheral blood samples, avoiding the need of repeat invasive biopsies^7^.

Moreover, while most cancer deaths are due to the haematological spread of metastases, research into the mechanism of metastasis initiation, formation, and propagation has been hampered by limited access to cancer cells within the various stages of the metastatic cascade. CTCs additionally provide a unique window into the biology of cancer as it spreads through the blood stream. The rarity of CTCs compared to normal blood cells has provided significant technical challenges in developing isolation methods which are sufficiently sensitive and specific^8^.

CTC culture provides an expanded cell population for expression analysis, functional assays, and drug sensitivity testing^9,10^, and long-term primary CTC cultures provide an ideal laboratory tool for the investigation of the biology of metastasis formation^11^. However, establishment of long-term primary CTC cell cultures has proved to be challenging. To date, despite intensive efforts, only several long-term CTCs cultures have been reported worldwide, including in colorectal^12^, breast^13^, and prostate^14^ cancer, all established with modest success rates, with 1–16% of blood samples producing stable cultures. To improve culture rates, initial expansion of the CTC population using xenotransplantation into immunodeficient mice prior to *in vitro* culture has been trialled^1,15^.

In this current work we describe establishment of two novel and distinct CTC cell lines derived from patients with metastatic gastroesophageal cancer.

## Results

### Establishment and validation of long-term *in vitro* CTC cultures from patients with metastatic gastroesophageal cancer

A total of 41 blood samples were processed for CTC enumeration, with 23 samples processed for culture using the optimised protocol (15 ml blood sample with negative selection using the CTC Enrichment Cocktail). CTCs were detected in 38/40 samples (93%) by EpCAM (epithelial cell adhesion molecule) based capture using the IsoFlux system (one specimen clotted and was not processed for CTC enumeration), with ≥10 CTCs found in 22 (54%) of the samples. Numbers of CTCs detected ranged from 0–150, with the mean number of CTCs 27.3 (summarised in Supplementary Table 1).

Long-term continuous CTC lines were established from two male patients using the optimised protocol (Table 1) and in both cases viable, relatively pure cultures were seen within 3 weeks, expanded rapidly, and have been maintained continuously for over 12 months to date. The first CTC line was established from patient 20 (cell line UWG01CTC), who had a low CTC count of 3 by EpCAM based capture despite widespread nodal and bone metastases. Patient 20 had a distal oesophageal/gastroesophageal junction carcinoma diagnosed on endoscopy in October 2015. He received concurrent chemoradiotherapy to the primary tumour and locoregional nodal disease as planned neoadjuvant treatment. Despite an excellent local response to chemoradiotherapy, he rapidly developed widespread metastatic disease including a dural metastasis causing spinal cord compression. At the time of CTC sampling he underwent resection of this metastasis, with histopathology demonstrating high grade neuroendocrine carcinoma, a rare and highly lethal subtype of cancer occurring in <1% of patients with gastrointestinal cancers^16^. Unfortunately, patient 20 progressed rapidly prior to receiving further treatment and passed away.

**Table 1:** Characteristics of the source patients of long-term CTC cell lines.

Patient Number/Cell line ID	Primary Tumour	Sites of metastatic disease at blood draw	Treatment prior to blood sampling	CTC count (IsoFlux, 7.5 ml blood)	Key protein expression of cell line
20/UWG01CTC	Distal oesophageal high grade neuroendocrine carcinoma	Widespread bone, nodal, and hepatic	Chemoradiotherapy with carboplatin and paclitaxel to primary tumour and regional nodal disease	3	Synaptophysin+ CGA+ CD56+ EpCAM−Cytokeratin weak/low
41/UWG02CTC	Gastric adenocarcinoma	Bone and peritoneal	Nil	109	EpCAM+ Cytokeratin+ CD44+

The second long-term culture was established from patient 41 (cell line UWG02CTC) who had a high CTC count of 109. This patient presented with diffuse bone and peritoneal metastasis. Endoscopy demonstrated a large ulcerated gastric mass confirmed on biopsy to be a gastric adenocarcinoma. A matched culture was established simultaneously from the ascitic fluid sample from the same patient (UWG02ASC). Patient 41 also progressed rapidly prior to receiving any anti-neoplastic treatments and passed away.

**Figure 1:**
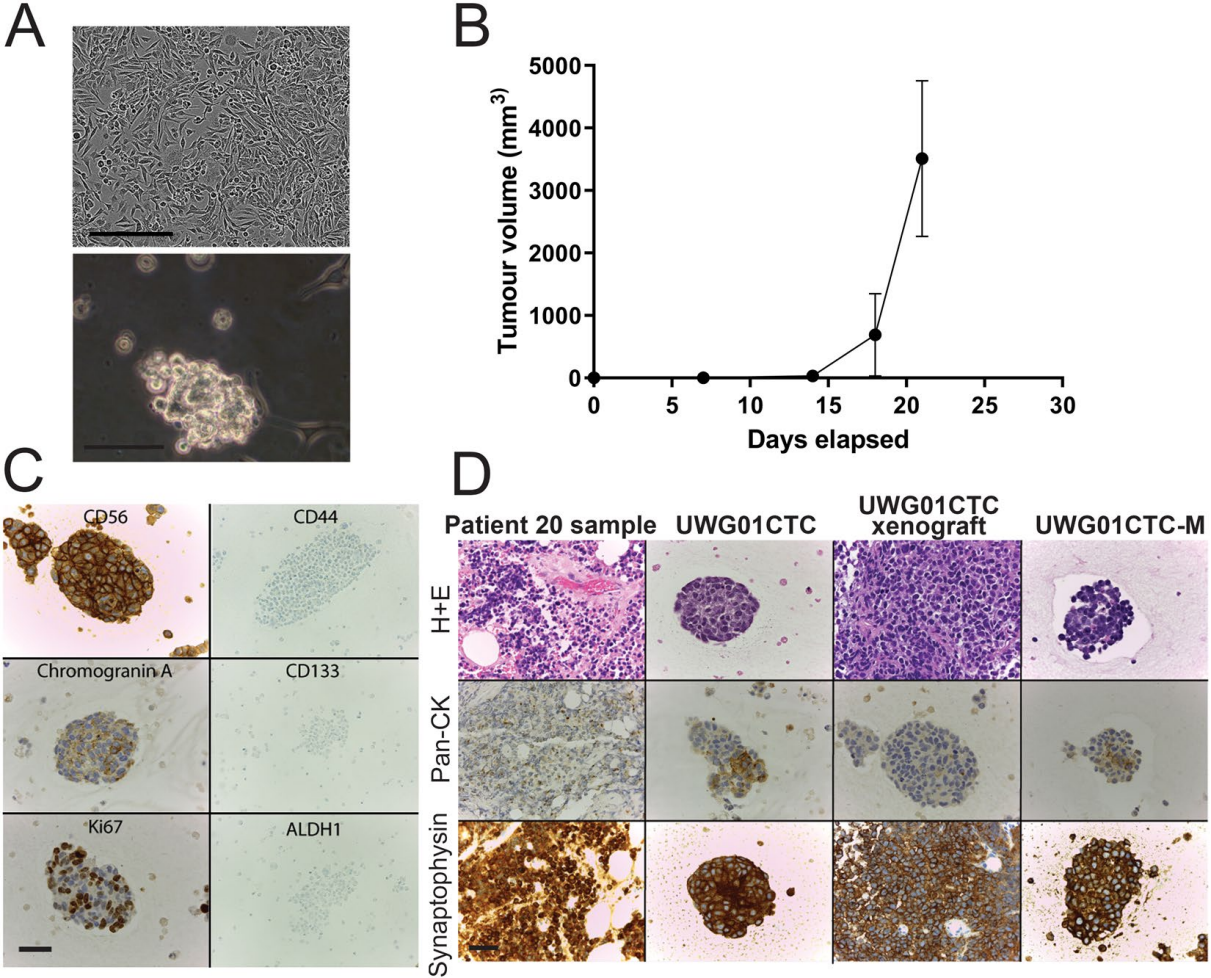
Characteristics of patient 20 tumour and UWG01CTC cell line. (**A**) Representative images of late passage (passage 40) UWG01CTC under hypoxic conditions in standard culture vessels with 10% FCS containing media (top image) or serum free media where they form loose spheroids (bottom image). Scale bar 50 µm. (**B**) UWG01CTC rapidly formed tumours in immunocompromised mice (n = 3), with all tumour endpoints reached within 3 weeks. (**C**) IHC analysis of UWG01CTC showing strong expression of neuroendocrine markers (CD56 and CGA), high Ki67 expression, but no expression of CSC markers (CD44, CD133, ALDH1). (**D**) IHC analyses of patient 20 tumour, patient derived cell line, mouse xenograft, and cell line derived from mouse xenograft (UWG01CTC-M) showing stable strong expression of the neuroendocrine marker synaptophysin, with consistent patchy cytokeratin positivity. Scale bar 100 µm.

**Figure 2:**
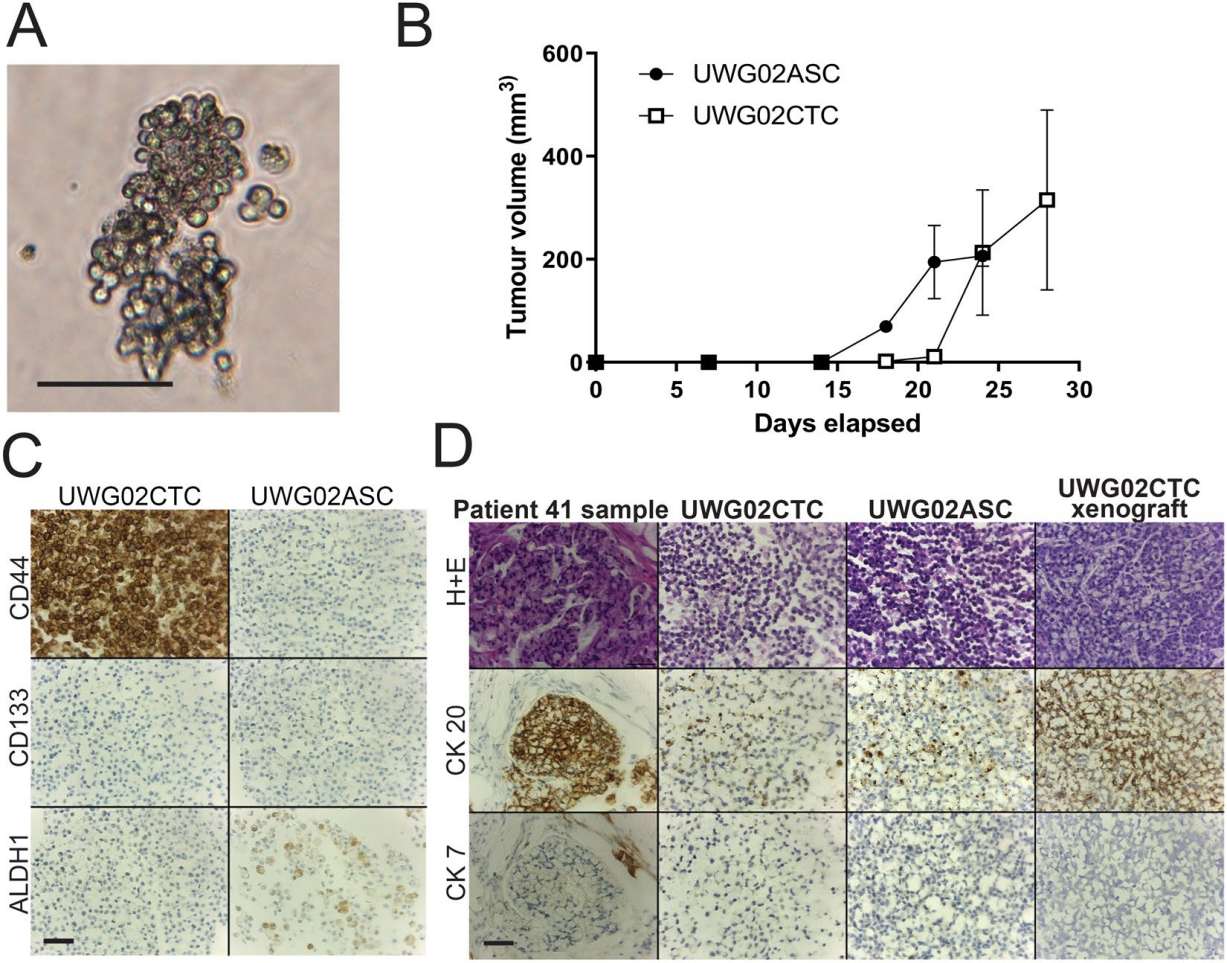
Characteristics of patient 41 tumour, UWG02CTC and UWG02ASC cell lines. (**A**) Representative images of the loose aggregates formed by UWG02CTC. Scale bar 50 µm. (**B**) Both UWG02CTC (n = 3, open squares) and UWG02ASC (n = 2, solid dots) rapidly formed tumours in immunocompromised mice, with all mice reaching tumour endpoints within 4 weeks. (**C**) Expression of cancer stem cell markers in UWG02CTC and UWG02ASC. Scale bar 100 µm. (**D**) IHC analyses of primary tumour, UWG02CTC and UWG02ASC. Both cell lines showed strong CK-20 and weak CK-7 staining, with an identical expression profile in tumours formed in the mouse xenograft. Scale bar 100 µm.

The CTC lines display discrete *in vitro* growth characteristics. UWG01CTC is adherent and requires trypsinisation for passaging, although a loose adherent spheroid phenotype is inducible in a hypoxic environment and serum free media (Fig. 1A). In contrast, both UWG02CTC (Fig. 2A) and UWG02ASC (data not shown) grow in long mucinous, loosely aggregated and weakly adherent strands which requires only gentle mechanical dissociation for passaging. All established cell lines have been adapted to grow in a variety of conditions, including serum free media supplemented with various growth factors, normoxic atmosphere, or ultra-low attachment (ULA) or standard culture vessels, and remain viable after freezing and thawing at various passages.

**Figure 3:**
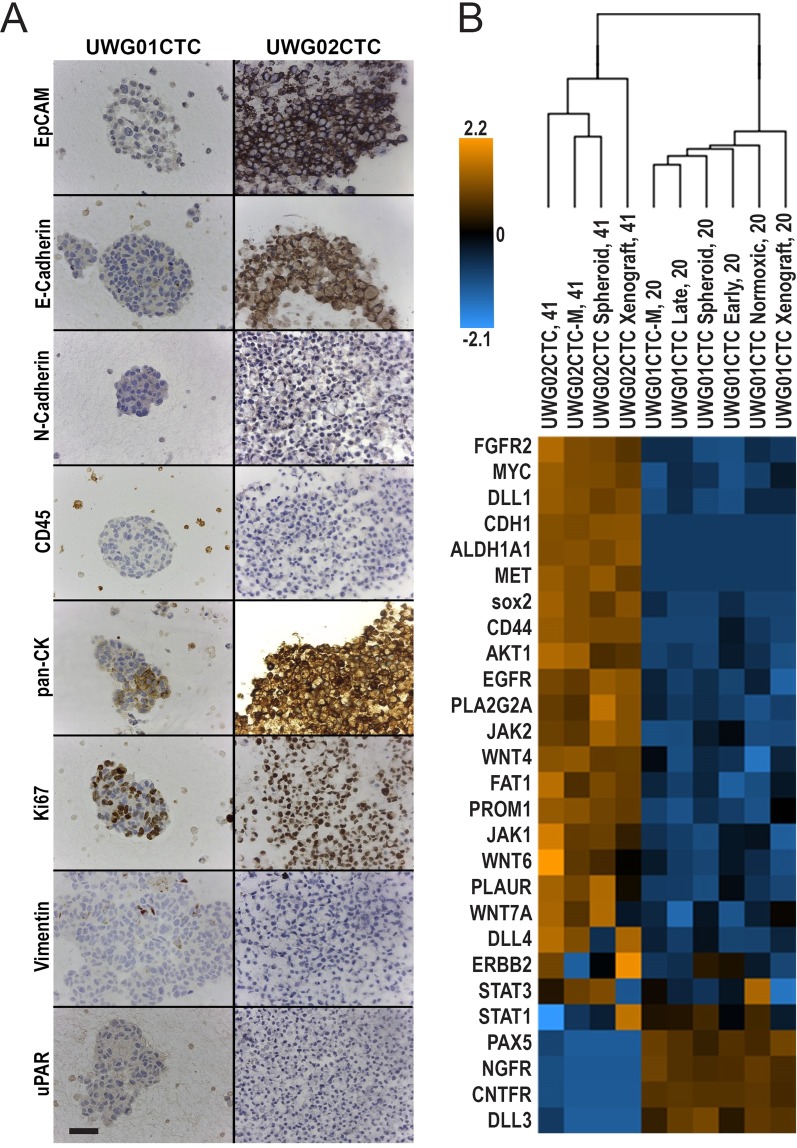
Comparison of UWG02CTC versus UWG01CTC. (**A**) Immunohistochemical analysis of the expression of key proteins. The distinct phenotypes of the two CTC cell lines are highlighted in this figure; UWG02CTC expresses epithelial markers (EpCAM and E-Cadherin) as well as strong cytokeratin staining. In contrast UWG01CTC has no epithelial staining and weak/patchy cytokeratin staining. Both cell lines show high Ki67 expression supporting a high proliferation rate. No CD45 staining was seen in any of the cell lines. Scale bar 100 µm. (**B**) Heat-map (unsupervised hierarchical clustering) of selected gene expression profiling of CTC cultures (UWG01CTC versus UWG02CTC) with matching mouse xenografts. Count data has undergone z-score transformation to give equal mean and variance across the samples. Orange indicates a higher score, blue indicates a lower score.

When injected subcutaneously into flanks of immunodeficient mice, both CTC lines rapidly formed tumours in all mice (n = 3 for each cell line), reaching tumour size endpoints (>10 × 10 mm) within 4 weeks for UWG01CTC (Fig. 1B) and 3 weeks for UWG02CTC (Fig. 2B). UWG02ASC also rapidly formed tumours (Fig. 2B). IHC on excised tumours confirmed identical expression of human cytokeratins and cell surface protein markers to the original patient tumour and corresponding cell line (Figs. 1D and 2D). Cell cultures were also successfully derived from mouse xenografts (denoted UWG01CTC-M, UWG02CTC-M and UWG02ASC-M to indicate passage through mice).

### Cell line authentication

Detailed DNA analyses were limited by poor DNA quality from both source patients’ formalin fixed paraffin embedded (FFPE) samples. However, by STR analysis, 24/28 (85.8%) alleles of UWG02ASC and UWG02CTC were identical to those of the primary tumour from patient 41, confirming the source of the cell lines (see Supplementary Table 2). Despite multiple attempts, there was inadequate DNA extracted from patient 20 for any analyses including STR. However, the STR profile of UWG01CTC did not match any known cell lines in the American Type Culture Collection (ATCC) or Deutsche Sammlung von Mikroorganismen und Zellkulturen (DSMZ) databases, confirming that UWG01CTC is a novel high grade neuroendocrine CTC line.

Further, UWG01CTC and UWG02CTC were analysed by targeted next generation sequencing and exhibited characteristic cancer gene amplifications or mutations for high grade neuroendocrine^17,18^ and gastric adenocarcinomas^19^, respectively (Supplementary Table 3).

### CTC derived cell lines recapitulate the pathological characteristics of source patients

Patient 20 primary tumour, UWG01CTC and xenograft demonstrated high grade poorly differentiated appearances by Hematoxylin and Eosin (H + E) stain (Fig. 1D) with a high Ki67 (>80%) (Fig. 1C). UWG01CTC displayed only weak patchy cytokeratin staining (Fig. 1D), but as expected expressed high levels of the neuroendocrine markers synaptophysin, CD56, and chromogranin A (Fig. 1C,D) as is typical of high grade gastroesophageal neuroendocrine tumours. Protein expression of synaptophysin was constant from cell line to mouse xenograft and subsequent cell culture propagated from the xenograft (UWG01CTC-M, Fig. 1D). UWG01CTC and its derivatives had an otherwise negative IHC profile, with no staining for epithelial or stem cell markers (Figs. 1C and 3A).

The primary tumour of patient 41 and UWG02CTC both demonstrated high grade appearances by H + E stain (Fig. 2D) with a high Ki67 (>80%) (Fig. 3A). As expected in adenocarcinoma, both the tumour and cell line strongly expressed cytokeratins (pancytokeratin; Fig. 3A), in particular CK-20 (Fig. 2D). UWG02ASC displayed an almost identical phenotype to UWG02 CTC (Fig. 2D, Supplementary Figure 1) except for the gastric cancer stem cell marker CD44, which was strongly positive in UWG02CTC but negative in UWG02ASC (Fig. 2C).

Differing media (10% FCS or serum free media) did not change the phenotype of any cell line as detected by IHC (data not shown). Neither CTC cell line expressed mesenchymal markers (vimentin or N-cadherin) or urokinase plasminogen activator receptor (uPAR), a key receptor for the initiation of proteolytic cascades^20^ by IHC (Fig. 3A). UWG02CTC strongly expressed EpCAM and E-cadherin proteins (Fig. 3A). No cell line showed any CD45 expression at any stage confirming these cultures did not derive from lymphocytes (Fig. 3A).

Expression of key IHC markers was maintained in each cell line in monolayer culture through to xenograft, and return to *in vitro* culture. These include neuroendocrine markers in UWG01CTC such as chromogranin A, CD56, and synaptophysin (Fig. 1C,D), and cytokeratin markers seen in adenocarcinomas in UWG02CTC (Supplementary Figure 1). No significant impact of varying *in vitro* culture conditions such as O_2_ levels, passage number, growth as a spheroid, and post xenograft culture compared to *in vivo* (xenograft) growth was observed on NanoString gene expression profiles using unsupervised hierarchical clustering, though monolayer (2D) cultures grouped together and away from spheroids (3D) and xenografts, which grouped together (Supplementary Figures 2 and 3A). While all patient 41 derived cultures and xenografts grouped away from the source tumour, global significance scores indicated that most gene sets (of key cancer pathways) genes exhibited little differential expression between UWG02CTC/ASC xenografts and the source tumour (Supplementary Figure 3B). Differential expression became more extensive as the culture models went from 3D to 2D compared to the source tumour (Supplementary Figure 3B). The CTC and ASC derived cultures and xenografts grouped closely together in all unsupervised clustering analyses with no significant gene expression differences observed confirming their common tumour of origin (Supplementary Figure 3A).

Thus the two established CTC cell lines not only demonstrate markedly different phenotypes that faithfully recapitulate those of their source tumours (Figs. 1–3), but also maintenance of their phenotype under the various *in vitro* and especially *in vivo* conditions tested, indicating their phenotypic stability.

### CTC cell lines have distinct gene expression profiles

The IHC and STR findings, showing two highly distinct CTC lines, were further explored by NanoString analyses. As expected the sample cohorts derived from patient 20 versus patient 41 grouped together by unsupervised hierarchical clustering (Supplementary Figure 4A). The majority of genes included in this NanoString gene panel were significantly differentially expressed between Patient 20 derived CTC cultures and xenografts compared to Patient 41 derived CTC cultures and xenografts (Supplementary Figure 4B). We then compared the expression of a subset of genes known to distinguish neuroendocrine from adenocarcinoma tumours using the CTC derived cultures and xenografts only. As expected UWG01CTC had higher expression of genes for neuroendocrine markers including *CNTFR, PAX-5 and NGFR*^21–23^ (Fig. 3B). UWG02CTC had higher expression of *CDH1* (encoding E-cadherin) relative to UWG01CTC (−log_2_ 9.4 fold). Interestingly, UWG02CTC when compared to UWG01CTC, had a higher expression of genes known to be involved in Helicobater pylori mediated carcinogenesis such as *AKT*^24^, *ETS2*^25^ and *MYC*^26^ (Fig. 3B), supporting endoscopic finding that patient 41’s tumour was likely related to *H. pylori* gastritis. There was also upregulation of genes encoding for the gastric cancer stem cell markers *CD44*, *ALDH1* and *CD133*, as well as key stem cell pathways such as *NOTCH* and *WNT*., These included the notch ligand delta-like ligands (DLL-1 and DLL-4) and PLA2GA, an important regular of metastases in gastric cancer and expressed with constitutively active Wnt^27–29^. UWG02CTC also showed higher expression than UWG01CTC of targetable pathways including *EGFR, FGFR2, HER-2 (ERBB2)*, and *MET*, as well as key genes in the JAK/STAT pathway, genes which overexpression are frequently reported in gastric adenocarcinomas but not gastrointestinal neuroendocrine cancers^16,30,31^.

UWG01CTC showed a higher expression of *DLL-3*, which is known to be expressed on most high grade neuroendocrine cancers, particularly small cell lung cancer. Compared to UWG02CTC, UWG01CTC also showed a lower expression of key DNA repair kinases, including *ATM* and *ATR*. We did not observe other reported molecular features of high grade neuroendocrine cancers such as *BCL-2* overexpression or Rb inactivation.

We did not find overexpression of CSC or epithelial to mesenchymal transition (EMT) genes in UWG02CTC compared to UWG02ASC and overall these two Patient 41 lines demonstrated very similar gene expression profiles.

### The CTC lines display differential drug and radiosensitivity profiles

**Figure 4:**
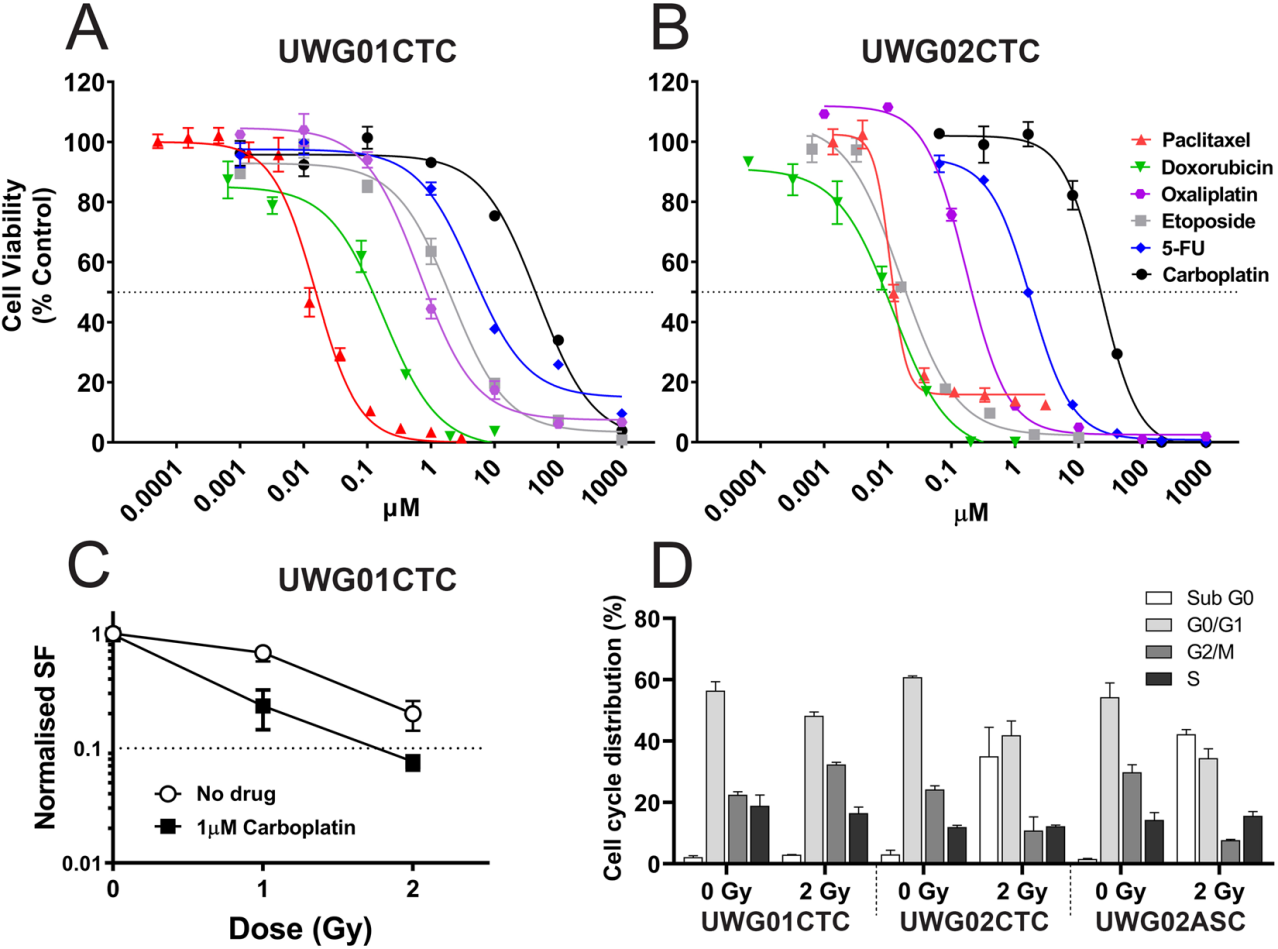
Drug responsiveness of CTC cell lines. Representative dose response curves for cytotoxic agents against (**A**) UWG01CTC and (**B**) UWG02CTC. All analyses were performed in hypoxic conditions. Cell viability of treated cells was normalised against vehicle controls, and presented as mean ± SEM (n = 3). 50% viability for IC_50_ is indicated by the grey dotted line. (**C**) Cell survival of UWG01CTC determined by clonogenic assay. Cells were irradiated with or without carboplatin (1 μM). Surviving fractions of irradiated cells only (no drug) and drug only were normalized to unirradiated non drug treated control. Each data point represents the means ± SEM of at least two independent experiments. (**D**) Comparison of cell cycle distribution for each cell line following radiotherapy. Each cell line has been normalised to the matched untreated control.

The *in vitro* sensitivity of both CTC and UWG02ASC cell lines to commonly used DNA-damaging chemotherapeutics with varying mechanisms of action was evaluated. UWG01CTC and UWG02CTC varied in their sensitivities to these drugs as demonstrated by the dose response curves shown in Fig. 4A,B. UWG01CTC was significantly less sensitive than UWG02CTC to all drugs including carboplatin and paclitaxel which patient 20 received prior to sampling for CTC culture (Table 2). UWG02CTC was 30–40× more sensitive to both doxorubicin and etoposide than UWG01CTC (Table 2). Indeed, UWG02CTC IC_50_ value for etoposide at 0.036 µM is ~3-fold lower than the minimum value reported for other gastroesophageal cancer cell lines (0.136 µM)^32^, suggesting that patient 41 may have benefited from treatment with this drug even though etoposide is more commonly used for neuroendocrine tumours. The IC_50_ values for the other compounds otherwise fall within the ranges reported for other cancer cell lines^32^. Expectedly, UWG02CTC and UWG02ASC cell lines were very similar in their drug sensitivities (Supplementary Table 4).

**Table 2:** Drug IC_50_ values for UWG01CTC and UWG02CTC (from P8 – 40^a^; all hypoxic conditions).

Drugs	UWG01CTC	UWG02CTC
	IC_50_ (µM)^b^	
Carboplatin	40.80 ± 2.94 (n = 6)	30.50 ± 8.50 (n = 4)
5-Fluorouracil	8.12 ± 3.40 (n = 3)^c^	1.51 ± 0.24 (n = 5)
Doxorubicin	0.64 ± 0.45 (n = 2)	0.02 ± 0.009 (n = 3)
Etoposide	1.57 ± 0.40 (n = 3)^d^	0.04 ± 0.021 (n = 3)
Oxaliplatin	0.93 ± 0.26 (n = 2)^e^	0.18 ± 0.052 (n = 6)
Paclitaxel	0.02 ± 0.01 (n = 5)	0.01 ± 0.003 (n = 3)

^a^From establishment of pure cell line. ^b^Mean ± SEM (of n separate experiments as shown). ^c^P = 0.0379 compared to UWG02CTC. ^d^P = 0.0209 compared to UWG02CTC. ^e^P = 0.0029 compared to UWG02CTC.

**Figure 5:**
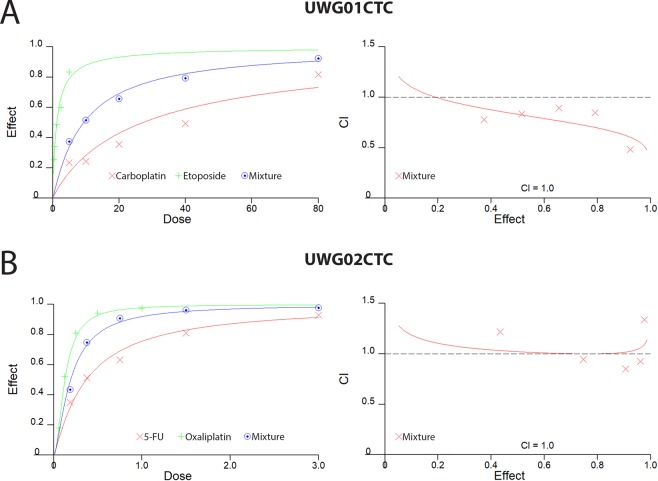
Combination drug treatment. (**A**) UWG01CTC and (**B**) UWG02CTC dose-effect curves and Fa-CI plots simulated by the median-effect equation for two drugs shown using CalcuSyn software with actual single drug and combination drug data points shown. Representative of at least 2 separate experiments each performed in triplicate.

The effect of combination drug treatment of the CTC cell lines was also explored. The Fa-CI plot (Fig. 5A) shows slight synergism for UWG01CTC at all concentrations of carboplatin and etoposide as all the data points fell below CI = 1 (average CI value = 0.765). At high Fa (i.e. >90% of cells killed) however, synergism was strong (CI = 0.485) (Fig. 5A, Supplementary Table 5A). Patient 20 may have benefited from treatment with this combination of drugs with dose reduction, as the calculated dose reduction index (DRI) for carboplatin and etoposide to achieve this synergistic effect was 5.0 and 3.5, respectively (Supplementary Table 5A). Oxaliplatin and 5-fluorouracil are routinely used in combination in the treatment of metastatic gastric adenocarcinomas however, when used against UWG02CTC, no synergism was evident with additive effects at middle range concentrations and a slight antagonism was observed for the lowest and highest concentrations of drug combination used (Fig. 5B).

### Low dose carboplatin pre-treatment enhances UWG01CTC radiosensitivity

The sensitivity of UWG01CTC line to radiotherapy with and without carboplatin sensitisation was investigated using the clonogenic survival assay. This cell line was radiosensitive with the mean surviving fraction after 1 Gy and 2 Gy being 0.67 and 0.18, respectively. The addition of low dose carboplatin (1 μM; 40-fold lower dose than IC_50_) significantly enhanced the radiosensitivity of cells resulting in decreased mean surviving fractions compared with cells exposed to radiation alone, especially after 1 Gy (Fig. 4C).

Unfortunately, UWG02CTC was unsuitable for this assay as it did not form the requisite colonies. Thus, the radiosensitivity of the CTC lines was also compared by flow cytometric analysis of cell cycle distribution 48 h post 2 Gy irradiation (Fig. 4D and Supplementary Figure 5). The fraction of UWG02CTC cells in all phases was substantially reduced after irradiation compared to control cells (0 Gy) with the majority appearing in sub G0/G1 indicating that the majority of cells underwent cell death after radiation treatment. In contrast, irradiation led to an increased fraction of UWG01CTC cells in G2/M indicating cell-cycle arrest with little evidence of cell death.

## Discussion

We report the establishment and characterisation of two novel long-term (>1 year) gastroesophageal cancer cell lines derived from CTCs. These two cell lines are genotypically and phenotypically distinct, reflective of differing tumour biology in the donor patients. Similar to other reported CTC lines, UWG02CTC displayed an epithelial phenotype, with strong expression of EpCAM and pan-cytokeratin. In contrast, UWG01CTC, isolated from a patient with neuroendocrine tumour, showed variable and weak cytokeratin (Cam5.2) immunostaining, with strong expression of neuroendocrine markers (CD56+, synaptophysin+, chromogranin A+). UWG01CTC had no detectable EpCAM, mesenchymal (NCAD, Vimentin), or cancer stem cell marker (CD44, CD133, ALDH1). This phenotype makes UWG01CTC a unique EpCAM negative CTC cell line.

Both CTC derived cell lines recapitulated the phenotype of the source patient’s tumour, highlighting that CTCs are a representative tumour source. The RNA expression profiling confirmed these data, with each cell line’s expression clustered with the source patient’s tumour and the corresponding mouse xenograft. Both CTC derived cell lines demonstrated rapid *in vitro* and *in vivo* growth with a high grade histological appearance supportive of a stem cell (CSC) phenotype. It was interesting to detect differences in CD44, ALDH1 protein and *STAT3* gene expression between the UWG02CTC and UWG02ASC, which probably reflects the different pathways of tumour cell dissemination. Of note, higher expression of CSC markers and key stem pathways was also found on UWG02CTC versus UWG01CTC, despite ALDH1, CD44, and CD133 reported as CSC markers in both adenocarcinomas and high grade neuroendocrine tumours^33,34^.

The preserved overall RNA expression profile and IHC staining pattern seen in tumours excised from the mice, and the cell cultures (UWG02CTC-M, UWG01CTC-M) derived from these tumours, highlight that these procedures, at least in short term cultures, do not grossly alter the original tumour characteristics, making CTC derived cell lines an excellent model to study tumour biology. Together with the confirmed tumourigenicity of the cell lines by the rapid development of tumours (within 4 weeks of inoculation) in all xenografted mice, and presence of key mutated and amplified genes that are characteristic of gastric adenocarcinomas or high grade neuroendocrine tumours, confirms the establishment of two novel CTC derived immortalised cell lines.

We also undertook cytotoxic and radiotherapy assays of the CTC cell lines based on standard cytotoxic agents used for these cancers. We found drug sensitivities were generally similar to those previously reported for gastroesophageal cell lines^35^. All cell lines were very sensitive to radiotherapy, with a synergistic effect of carboplatin seen in UWG01CTC. This is consistent with the clinical experience of the radiosensitivity displayed by high grade neuroendocrine cancers, driven by low expression of key DNA damage response enzymes, such as ATM and ATR^36–38^. We also explored potential synergistic effects of drug combinations as this may allow clinical dose reduction, which in turn reduces toxicity while retaining the therapeutic efficacy.

Multiple other potential therapeutic targets were identified by RNA expression profiling. UWG02CTC had high expression of *EGFR, FGFR2, ERBB2*, and *JAK/STAT* pathway genes, suggesting potential sensitivity to treatments directed at these validated targets, while UWG01CTC had high expression levels of the *DLL3* gene, which is proposed as a potentially druggable target^39^. While these data show that CTC derived cell lines can be used to define personalised drug sensitivities for gastric cancer patients, it is important to note the rapid disease progression and clinical decline of the patients that yielded CTC cultures. The test results in our study would have been too late to guide management, and faster protocols are needed to inform personalised therapy in a clinically meaningful way. Nevertheless, more comprehensive drug sensitivity testing will be performed on the established CTC lines, and may help to define better drug screening libraries for faster future protocols.

Other studies have suggested a high CTC count (>300) is necessary for successful culture^12^. While we agree with this requirement, this issue is complicated by the inherent biases when selecting the isolation method and epithelial marker based CTC definition used for enumeration. We employed the standard EpCAM based isolation and cytokeratin based CTC identification in the matched blood sample used for CTC enumeration. Due to the biological differences discussed above, our successful cultures derived from samples with high (109) and low (3) CTC counts, subsequently developing EpCAM positive and negative cell lines, respectively. This finding highlights a key issue in the CTC field. While patient 20 has a low EpCAM+ CTC count, which is within the range of background cells found to express CTC markers (i.e. false positive CTCs) detected from healthy donor blood samples with the IsoFlux system (data not shown), the successful establishment of a CTC cell line argues that this patient had a high number of EpCAM− CTCs with the ability to establish metastatic deposits. This hypothesis is also supported by the clinical picture. CTC numbers are known to increase with disease burden^40^, and at the time of blood draw patient 20 had high volume and rapidly progressive disease. It is reasonable to suggest this patient had a high number of biologically relevant CTCs which were missed using standard CTC isolation techniques. The negative selection used for CTC culture (CD45 depletion), rather than the positive selection used for CTC enumeration (EpCAM capture) was critical to detect these cells. The optimal isolation method of CTCs continues to evolve in the face of these issues, and while more emphasis has been given to epithelial to mesenchymal transition (EMT) phenotype CTCs in recent years^41^, the data presented here highlight the persistent challenges identifying the optimal markers to isolate CTCs.

Both CTC cell lines displayed a highly aggressive phenotype, with rapid growth, high grade histological appearance, and high Ki67 expression. In both cases, this phenotype mirrored the rapid clinical progression seen in the corresponding patient. Similar results have been seen in other CTC cell lines^12^. It is unclear whether this finding is indicative that a high proliferation rate is a requirement for successful cultures, or is a result of selection pressures for aggressive clones in the culturing process.

The matched culture derived from ascitic fluid (UWG02ASC) simultaneously established from patient 41 as UWG02CTC demonstrated an identical phenotype, with the exception of cancer stem cell marker expression. UWG02CTC was strongly positive for CD44, a key CSC marker in gastric cancer^28^, while UWG02ASC was negative for CD44. This is consistent with other results showing that CTC cultures exhibit a stem cell phenotype, and further supports the notion that CTC cultures develop from the CTC population which are able to establish metastases^12,32,42^.

In conclusion, we report the first two long-term CTC cell lines developed from patients with metastatic gastroesophageal cancer, providing a high fidelity platform for high-throughput gene expression, drug response profiling and functional genomics studies. The two CTC cell lines displayed distinct profiles which faithfully recapitulate the source patient’s tumour. Our results support the developing role of CTC culture as an essential laboratory resource for the understanding of the biology of metastases and importantly undertake personalised screening for therapeutic strategies.

## Methods

### Patient selection and blood collection

Peripheral venous blood samples were collected from patients with metastatic gastroesophageal cancer prior to treatment. Patients had histologically confirmed gastric or gastroesophageal cancer treated at the Illawarra Cancer Centre, Wollongong Hospital, NSW Australia. Informed consent was obtained from each patient prior to enrolment, and the study was approved by South Western Sydney Local Health District Human Research Ethics Committee (Project Number 15/072). All experiments were performed in accordance with relevant guidelines and regulations.

Initially 7.5 ml of blood was collected in EDTA tubes. This was increased to 15 ml after seventeen patients were enrolled for higher CTC capture to improve culture success rates. Blood samples were transported immediately at room temperature for CTC isolation. A separate matched 7.5 ml EDTA blood sample was collected at the same blood draw for EpCAM based capture for CTC enumeration using the IsoFlux system (Fluxion Biosciences), and processed as per manufacturer instructions^43^. Enumerated CTCs were defined by the standard EpCAM/Cytokeratin/DAPI positive and CD45 negative phenotype^40^.

### CTC isolation and cell culture

Blood samples were incubated for 20 min with RosetteSep CTC Enrichment Cocktail with anti-CD36 (Stemcell Technologies, Cat. 15167) prior to a density gradient separation with Leucosep tubes (Stemcell Technologies) to isolate a peripheral blood mononuclear cell (PBMC) layer. We initially found excessive lymphocyte contamination prohibiting CTC growth with the RosetteSep Human CD45 Depletion Cocktail alone. This improved with changing to the CTC Enrichment Cocktail. The PBMC layer was washed twice, then immediately plated into 24-well ultra-low attachment plates (Corning) with serum free Advanced DMEM/F12 (ADMEM/F12; Sigma Aldrich) supplemented with epidermal growth factor (EGF; Life Technologies), fibroblast growth factor (FGF; Life Technologies) and N2 supplement (Life Technologies), or ADMEM/F12 with 10% foetal calf serum (FCS) in hypoxic conditions. Separate CTC cultures were maintained in 10% FCS or serum free media. While serum free media is reported to support a cancer stem cell phenotype^44^, in both cell lines successful cultures were established in both media formulations, and we did not find any phenotypic differences between media conditions (data not shown). All media was supplemented with antibiotics (see Supplementary Table 6 for media formulations).

Patient 41 had marked peritoneal disease and gross ascites from gastric adenocarcinoma. At the same time as the blood draw for CTC isolation, 200 ml of ascitic fluid were collected from a peritoneal catheter. This sample was transferred immediately to the laboratory, centrifuged to collect a cell pellet, washed twice in ADMEM/F12 with 1% antibiotic/antimycotic (Sigma Aldrich), and cultured as per conditions listed above. Cultures derived from the patient’s CTC and ascites samples were maintained independently under identical conditions. Once established as cell lines, for all subsequent routine cell culture and experimental assays, cells were maintained in ADMEM/F12 with 10% FCS supplemented with EGF.

### Tumourigenicity of cell lines in mouse xenograft models

For confirmation of tumourigenicity, 1–2 × 10^6^ cells from early passages of each cell line were injected subcutaneously into the flank of NOD *scid gamma* (NOD.Cg-*Prkdc*^*scid*^ *IL2rg*^*tm1Wjl*^/SzJ) mice. Mice were monitored for tumour growth and sacrificed when the tumour grew greater than 10 × 10 mm, or the animal demonstrated signs of stress (such as >15% weight loss) or evidence of impedance of tumour on movement. Tumours were collected from the sacrificed mice for subsequent culture, RNA and DNA extraction, and histological analysis. For culture from xenografts, tumour tissues were cut into approximately 1 mm pieces and then incubated with tumour dissociation enzymes (Tumour dissociation kit, Miltenyi Biotec) in ADMEM/F12, after which the tumour homogenate was centrifuged, the pellet resuspended, and plated in serum-free media and hypoxic conditions as above. All procedures were carried out in accordance to the Australian Code for the Care and Use of Animal for Scientific Purposes 8^th^ edition 2013, and approved by the University of Wollongong’s Animal Ethics Committee (study AE15/17).

### Immunohistochemical analysis of patient samples, cell lines, and mouse xenografts

Expression of key proteins on the cell lines, mouse xenografts, and representative sections from the matching patient’s tumour specimen were compared using immunohistochemistry (IHC). For cell lines, cells were collected, centrifuged with supernatant removed, then clotted with plasma and commercially prepared thrombin (Fibri-Prest Automate from Stago) to prepare a cell block. All samples were fixed in 10% formalin and paraffin embedded, with 4 µm sections cut for staining. Antigen retrieval and development was performed on the fully automated Bond system according to manufacturer’s instructions (See Supplementary Table 7 for antibody details), with positive controls included on each slide.

### DNA and RNA extraction

Tumour, cell culture and xenograft nucleic acids were extracted using the AllPrep DNA/RNA FFPE Kit (80234, Qiagen) or AllPrep DNA/RNA/Protein Mini Kit (80004, Qiagen), respectively, according to the manufacturer’s instructions. All samples were quantified using the NanoDrop (ND1000, Thermo Fisher Scientific). DNA/RNA samples had A260/280 ratios between 1.8 and 2.1.

### NanoString analysis

Total RNA extracted from fresh-frozen (25 ng) and FFPE (150 ng) specimens were run on the NanoString nCounter Sprint system using the 770 gene PanCancer Pathways panel with additional cancer stem cell and proteolytic genes as per the manufacturer’s instructions (NanoString Technologies). RNA extracted from patient 20 FFPE specimen had low RNA integrity and did not meet the threshold for further analysis. Results were analysed using the NanoString nSolver 4.0 and Advanced Analysis Module, which normalizes gene expression to a set of positive and negative control genes built into the platform^45^. As recommended, genes with expression levels at or below the level of the negative controls were removed from analysis. Differential expression of key transcriptomic pathways were compared between cell lines, with fold change and *P* values calculated using nSolver default settings. With the remaining list of genes, a filter cutoff of fold change ≥ ±2 and *P* value < 0.05 were used to identify the significant gene expression changes^46^.

### Short Tandem Repeat and targeted gene sequencing analysis

STR profiles for the cell lines and their matching patient FFPE tumour tissue were verified by the PowerPlex 18D System, using the following 18 markers (seventeen STR loci and Amelogenin): D3S1358, TH01, D21S11, D18S51, Penta E, D5S818, D13S317, D7S820, D16S539, CSF1PO, Penta D, Amelogenin, vWA, D8S1179, TPOX, FGA, D19S433 and D2S1338. As per standard practice, cell lines were considered to match if profiles are more than 80% identical to source patient sample.

DNA of the cell lines and xenografts were sequenced using the Oncomine Comprehensive v3 Assay on an Ion S5 Sequencer (Thermo Fisher Scientific). The assay detects specific hotspots as well as coding sequences in particular genes. STR and Oncomine assays were performed by KCCG Sequencing Laboratory & Cancer Diagnostics, Garvan Institute of Medical Research, Sydney Australia.

### Cytotoxicity assays

Approximately 10,000 cells were seeded per well in triplicate into a 96-well plate 48–72 h prior to drug treatment. Cells were incubated with serial dilutions of each drug for 72 h with drug vehicle (water, 0.9% saline, or DMSO, depending on drug solubility) and the dilution kept constant across all drug concentrations and controls (final concentration of 0.2%). The viability of cells were assayed using CellTitre 96 Aqueous One Solution Cell Proliferation Assay (Cat # G3581, Promega Corporation, Fitchburg, Wisconsin, USA) using a SpectroMax 250 UV plate reader using SoftMax Pro software (Molecular Devices, Sunnyvale, California, USA). Cell viability of treated cells was normalized against vehicle controls. This data was analysed using a logarithmic sigmoidal dose–response curve using the variable slope parameter to determine IC_50_ (GraphPad Prism 6.0, GraphPad Inc.). Data is presented as a mean ± standard error of the mean (SEM) from ≥2 independent experiments.

For combination drug assays cells were seeded as above and then treated with the drug combinations specified. The drug concentrations were kept at a constant ratio based on their approximate IC_50_ values determined as monotherapies and ranged from 0.125×, 0.5×, 1×, 2× and 4× the IC_50_ of each drug, as optimal for the Chou-Talalay median-effect equation^47^. To determine synergism/antagonism, the combination index (CI) and dose response index (DRI) was calculated using CalcuSyn (Biosoft, USA), with Monte-Carlo simulation to assign confidence. By the Chou-Talalay method, a CI = 1 represents an additive effect, a CI value > 1 represents antagonism and a CI value < 1 represents synergism.

### Irradiation procedure and clonogenic survival assay

The sensitivity of the UWG01CTC cells to 10MV x-ray irradiation with drug pre-treatment was investigated using clonogenic survival as the radiobiological endpoint as previously described^48^. UWG02CTC and UWG02ASC were not suitable for this assay as they formed loose and small colonies. Briefly, cells were first acclimated for at least a week in normoxic conditions, then plated into 12.5 cm^2^ tissue culture flasks in regular cell culture media (as above) in order to reach ~60% confluency 3 days later. Cells were then pre-sensitized with 0, 1, 2 and 5 µM carboplatin for 48 h prior to exposure to 1 or 2 Gy X-Ray radiation delivered in a single fraction at room temperature. Cells were then seeded into triplicate tissue-culture petri dishes (100 mm × 20 mm Falcon BD; Pacific Laboratory Products) in 10 mL of ADMEM/F12 with 10% FCS, pencillin/streptomycin, EGF and L-glutamine, at different cell densities per dish (ranging from 1000 to 20000 cells per dish). After approximately 15 doubling times, petri dishes were washed with PBS and adherent cell colonies fixed and stained with a 1:3 crystal violet:ethanol solution. Colonies (≥50 cells/colony) were manually counted and presented as Mean Plating Efficiency (MPE; [number of colonies]/[number of cells plated]*100) and Surviving Fraction (SF; MPE of treatment group/MPE of control group) as previously described^49^. Unirradiated control samples (with and without drugs) were handled under the same conditions as the irradiated samples.

### Cell cycle analysis

Measurements of cell cycle distribution after irradiation were performed essentially as previously described^50^. Cells were irradiated in T12.5 cm^3^ flasks using a Varian 2100iX linear accelerator. Prior to irradiation flasks were completely filled with medium. Flasks were located at midpoint in a 23 cm thick Solid Water phantom (Gammex Inc, USA). Flasks were surrounded with Super-Flex bolus (Radiation Products Design Inc, USA) to ensure electronic equilibrium. Doses of 1 or 2 Gy were delivered using parallel opposed beam geometry, in a single fraction at room temperature. Briefly, cells (2.0 × 10^4^) were stained with PI master mix (100 μg/mL RNase A, 40 μg/mL propidium iodide and PBS pH 7.4) for 30 min at 37 °C. DNA content was then measured using a Becton Dickinson BD LSR II FACSort flow cytometer (BD Biosciences, USA) and the proportion of cells in G0/G1, S and G2/M phases of cell cycle were calculated on the basis of DNA distribution histograms using FlowJo software as previous described (V7.1, Tree Star Inc., USA)^51^.

### Statistical analysis

Apart from the NanoString analyses, all the experimental data were expressed as means ± SEM and statistically analyzed using GraphPad Prism (version 6.0, USA). For comparisons, two-tailed Student’s *t* test, Fisher’s exact test, one-way analysis of variance test, and Dunnett’s test were performed. *P* values < 0.05 were considered statistically significant.

## Supplementary information

Supplementary Information.

## Data Availability

The datasets generated during and/or analysed during the current study are available from the corresponding author on reasonable request.
