# Supplementary material for: Establishment of novel long-term cultures from EpCAM positive and negative circulating tumour cells from patients with metastatic gastroesophageal cancer
Source: Sci Rep. 2020 Jan 17;10:539. doi: 10.1038/s41598-019-57164-6 (PMC6968999; doi:10.1038/s41598-019-57164-6)
Supplement: Supplementary file 1 — Supplementary Information. [file 41598_2019_57164_MOESM1_ESM.docx]

**Establishment of novel long-term cultures from EpCAM positive and negative circulating tumour cells from patients with metastatic gastroesophageal cancer.**

**Supplementary data**

Daniel Brungs^1, 2, 3, 4#^, Elahe Minaei^1,2,4^, Ann-Katrin Piper^1,2,4^, Jay Perry^1,2,4^, Ashleigh Splitt^3,4^,

Martin Carolan^1,3,4^, Shantay Ryan^5^, Xiao Juan Wu^5^, Stéphanie Corde^1,8,9^, Moeava Tehei^1,4,9^, Morteza Aghmesheh^1,3,4^ , Kara L. Vine^1,2,4^ , Therese M. Becker^4, 6 ,7^, Marie Ranson^1,2,4#^

^1^ Illawarra Health and Medical Research Institute, Wollongong, Australia

^2^ School of Chemistry and Molecular Bioscience, University of Wollongong, Wollongong, Australia

^3^ Illawarra Cancer Centre, Wollongong Hospital, Wollongong, Australia

^4^ CONCERT-Translational Cancer Research Centre, New South Wales, Australia

^5^ NSWHP Anatomical Pathology Liverpool Hospital, Liverpool Hospital

^6^ School of Medicine, University of Western Sydney, Liverpool, Australia

^7^ Centre for Circulating Tumour Cell Diagnostics and Research, Ingham Institute for Applied Medical Research, Liverpool Australia

^8^ Radiation Oncology Department, Prince of Wales Hospital, Randwick, Australia

^9^ Centre for Medical Radiation Physics, University of Wollongong, NSW, Australia

**Supplementary Figure 1:**

**
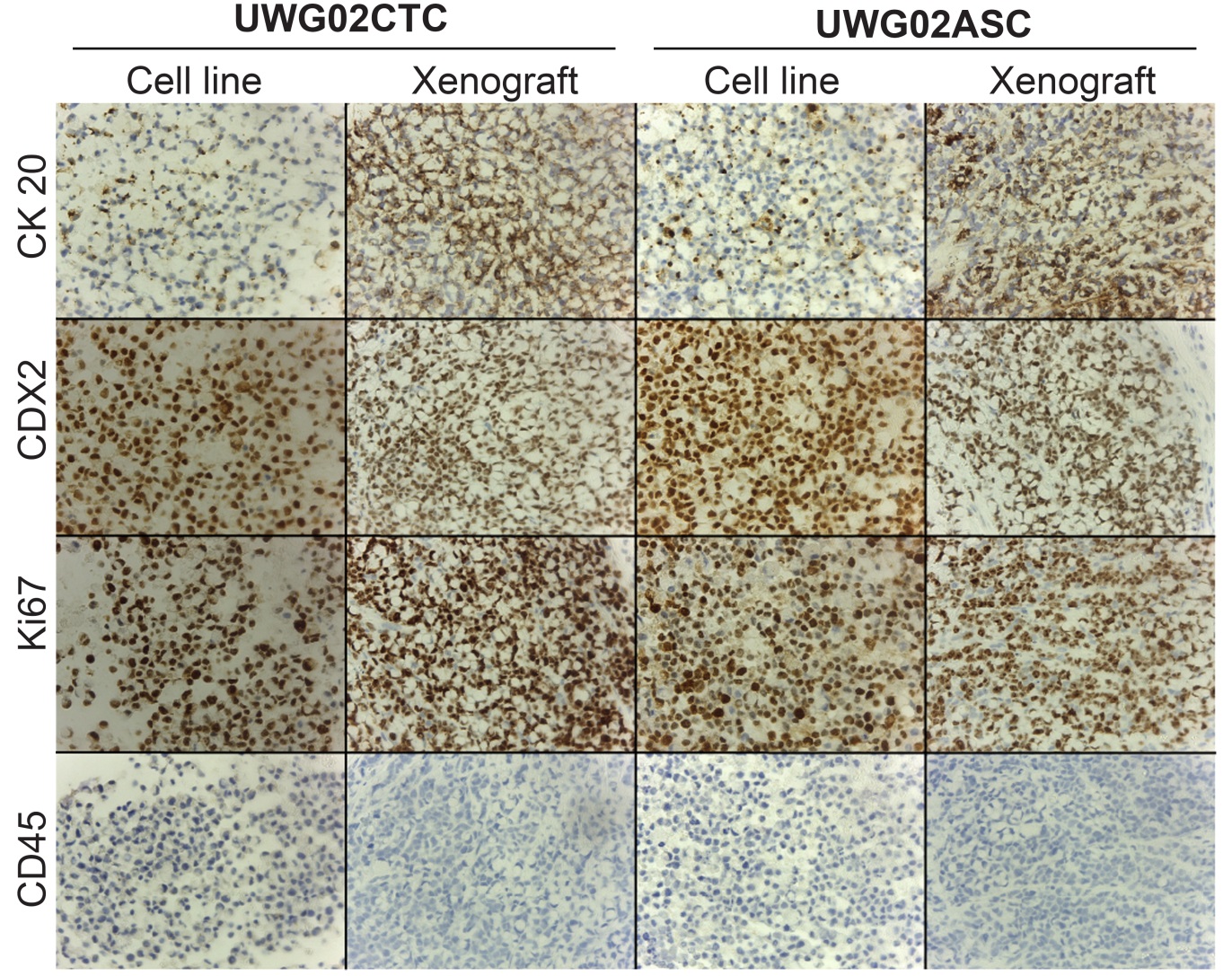
**

**Supplementary Figure 1:** UWG02CTC /ASC IHC analyses. Preservation of key IHC markers from early passage UWG02CTC or UWG02ASC as monolayer cultures to xenograft s in mouse.

**Supplementary Figure 2:**


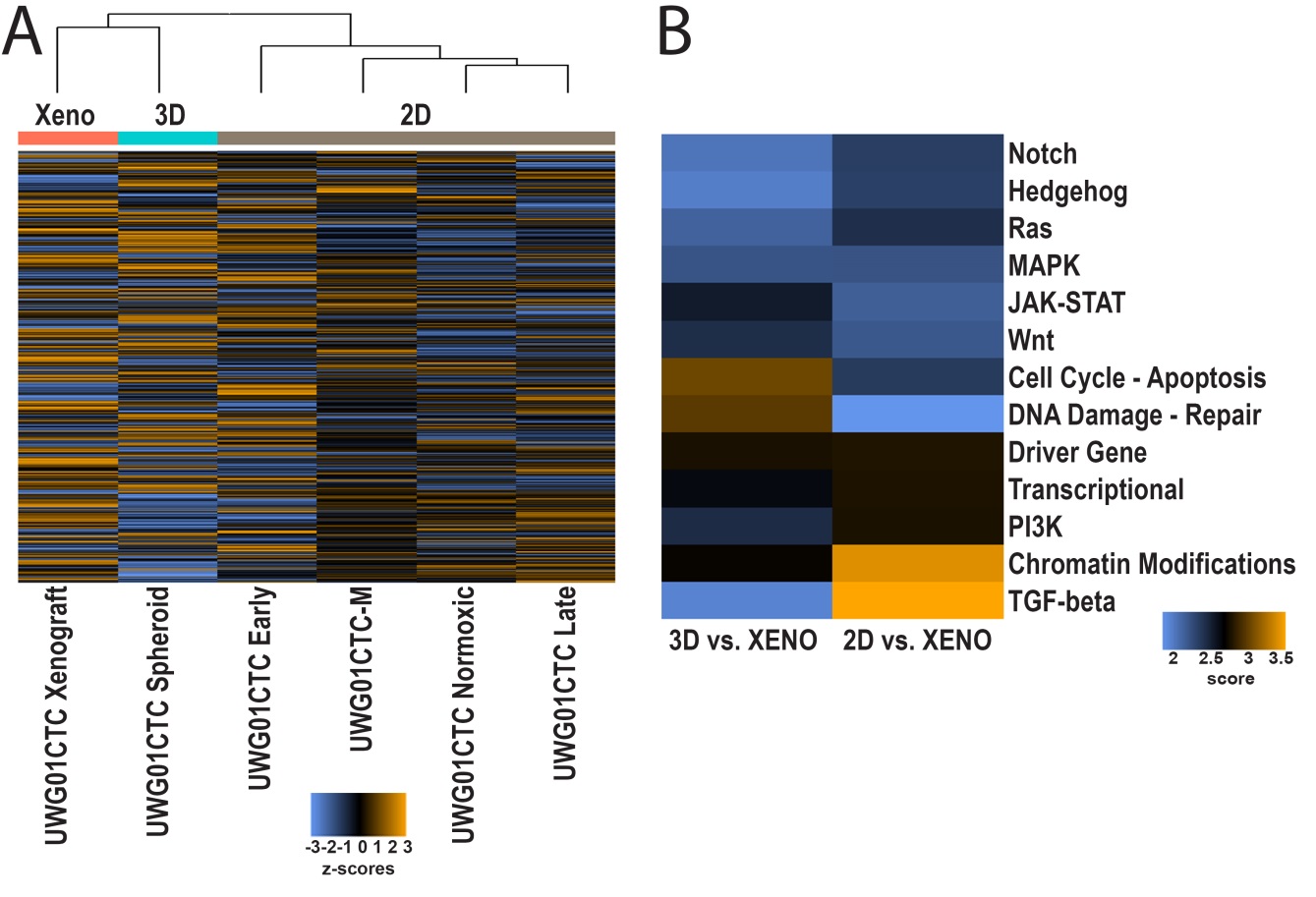


**Supplementary Figure 2:** UWG01CTC gene expression analyses. (A) NanoString heat-map of the normalized data (unsupervised hierarchical clustering, scaled to give all genes equal variance via z-score transformation), showing high-level overview of cancer gene expression (Pancancer progression panel) in patient 20 derived cultures and xenograft. Xenograft XENO), spheroid culture (3D) and various monolayer culture conditions (2D) were selected as covariates. Orange indicates high expression; blue indicates low expression. Horizontal columns represent individual genes (log2 count of 770 gene codeset), vertical columns represent individual samples. Complete sample identifiers are shown at the bottom. The sample tree was split at 3 levels with UWG01CTC growth as a spheroid (3D) and xenograft grouping together and the monolayer (2D) cultures grouping together but with UWG01CTC as an early passage (< 5) monolayer culture clearly grouping away from long term cultures or cells grown under normoxic conditions.

(B) Global Significance Scores, measures the extent of differential expression of a gene set’s genes with a covariate, ignoring whether each gene within the set is up- or down-regulated. Orange denotes gene sets whose genes exhibit extensive differential expression with the covariate, blue denotes gene sets with less differential expression. 2D – all monolayer cultures grouped together as a single cohort. There is less differential gene expression between the spheroid versus the xenograft sample than between monolayer cultures and the xenograft, with this being most pronounced in genes in chromatin modification and TGFB pathways.

**Supplementary Figure 3:**


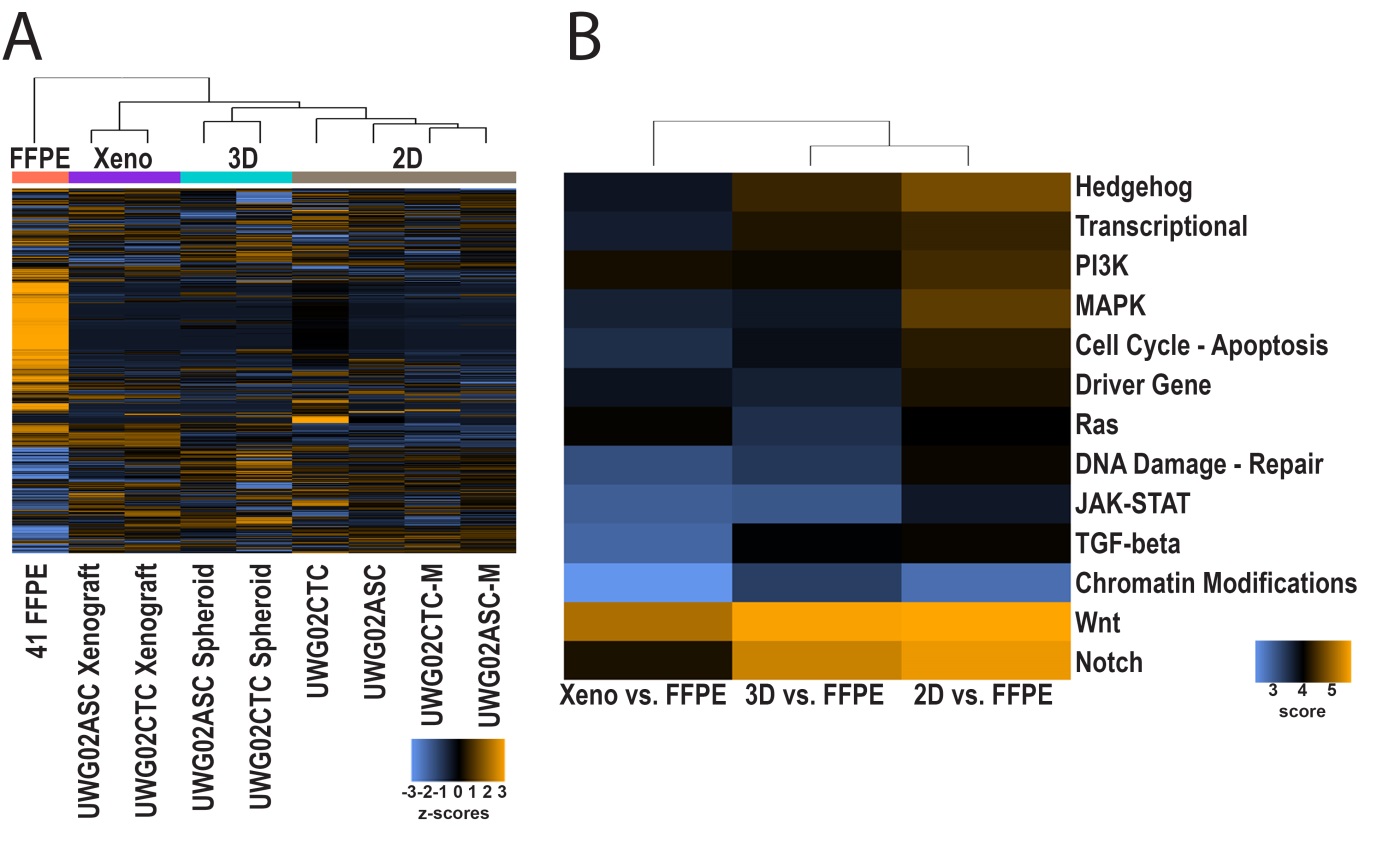


**Supplementary Figure 3:** UWG02CTC /ASC gene expression analyses. (A) NanoString heat-map of the normalized data (unsupervised hierarchical clustering, scaled to give all genes equal variance via z-score transformation), showing high-level overview of cancer gene expression (Pancancer progession panel) in patient 41 derived cultures and xenograft. Source tumour (FFPE), xenograft (XENO), spheroid culture (3D) and various monolayer culture conditions (2D) were selected as covariates. Horizontal columns represent individual genes (log2 count of 770 gene codeset), vertical columns individual samples. (B) Global Significance Scores. Orange denotes gene sets whose genes exhibit extensive differential expression with the covariate, blue denotes gene sets with less differential expression. 2D – all monolayer cultures grouped together as a single cohort.

**Supplementary Figure 4:**

**
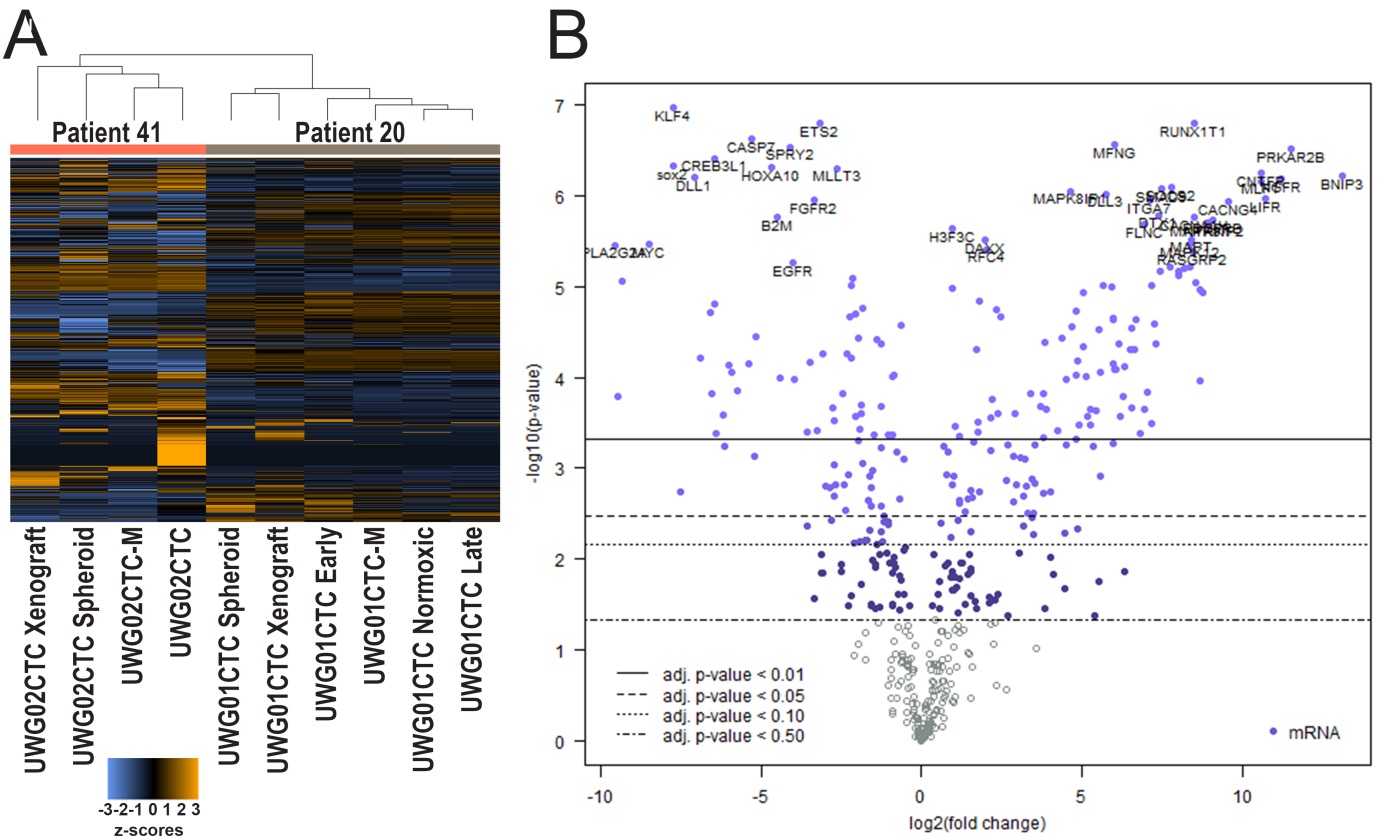
**

**Supplementary Figure 4:** Gene expression analysis comparison between Patient 41 and 20 derived CTC cultures and xenografts. (A) NanoString heat-map of the normalized data (unsupervised hierarchical clustering, scaled to give all genes equal variance via z-score transformation), showing high-level overview of cancer gene expression (Pancancer progression panel) in patient 20 versus patient 41 derived cultures and xenograft. Patient ID was selected as the covariate. Orange indicates high expression; blue indicates low expression. This plot provides a high level exploratory view of the data. Horizontal columns represent individual genes (log2 count of 770 gene codeset), vertical columns individual samples. (B) Volcano plot displaying each gene's -log10 (p-value) and log2 fold change with patient 41 as the covariate (i.e., patient 20 derived 2D and 3D models combined as a cohort versus baseline of patient 41 derived 2D and 3D models. Highly statistically significant genes fall at the top of the plot, and highly differentially expressed genes fall to either side. Gradient-colored points and horizontal lines indicate various adjusted p-value thresholds. The 40 most statistically significant genes are specifically labelled.

**Supplementary Figure 5:**

**
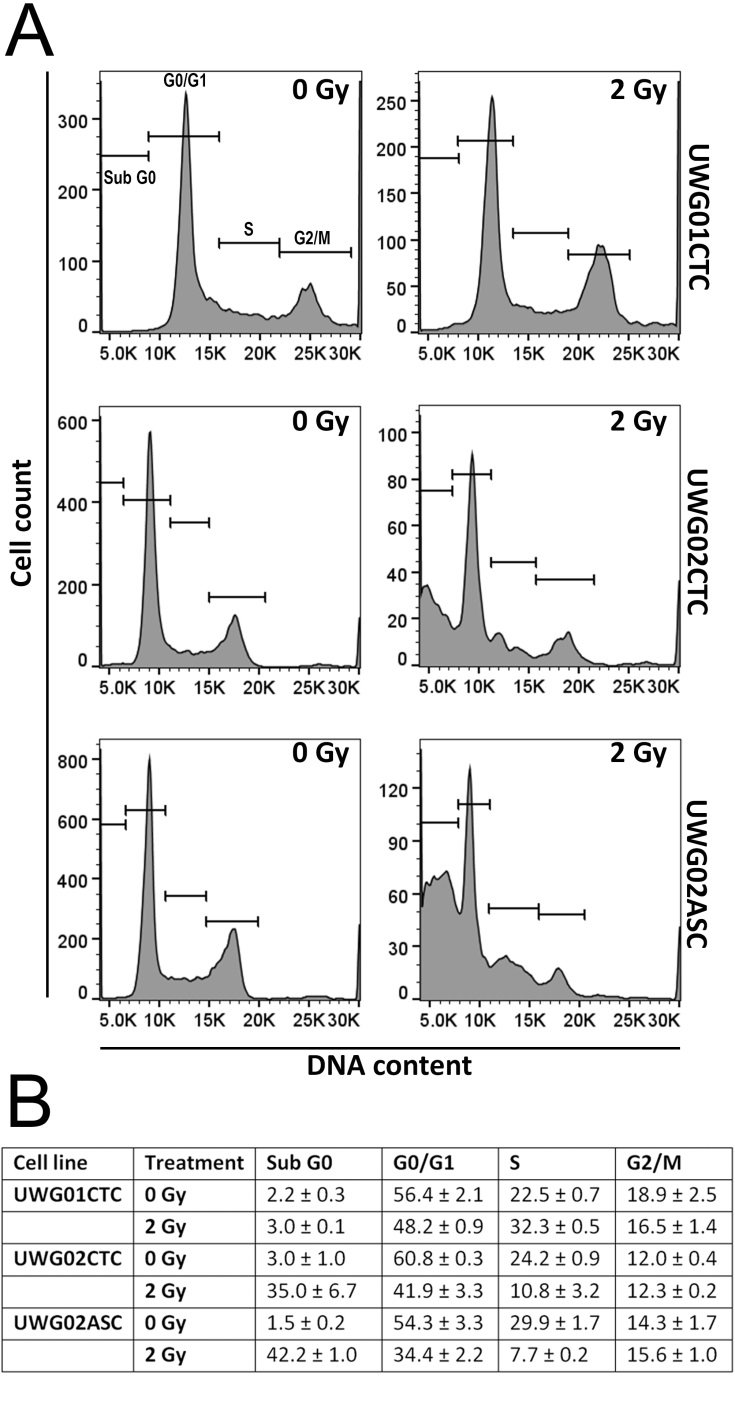
**

**Supplementary Figure 5:** Cell cycle analyses post radiation treatment of UWG01CTC, UWG02CTC and UWG02ASC. (A) Flow cytometric analysis of cell cycle stages using Propidium iodide (PI) as a nuclear stain. 48 h post radiation, UWG01CTC cells show 10% increase of the cell population in the G2/M phase (toward the right of the x-axis) indicating an arrested state for DNA damage control. UWG2CTC and UWG02ASC cells did not tolerate the 2 Gy-radiation which is reflected in the reduced cell numbers post radiation and an increased cell population found in a sub-G0 population (toward the left of the x-axis). (B) Table of results from two individual experiments performed in duplicate repeats.

**Supplementary Table 1**: Patient samples used for CTC culture

| Patient number | CTC count (Isoflux, 7.5ml blood) | Blood volume for ctc culture [ml] | CTC enrichment cocktail | Long term CTC culture established |
| --- | --- | --- | --- | --- |
| 1 | 150 | 7.5 | CD45 only |  |
| 2 | 0 | 7.5 | CD45 only |  |
| 3 | 38 | 7.5 | CD45 only |  |
| 4 | 27 | 7.5 | CD45 only |  |
| 5 | 12 | 7.5 | CD45 only |  |
| 6 | 4 | 7.5 | CD45 only |  |
| 7 | 2 | 7.5 | CD45 only |  |
| 8 | 26 | 7.5 | CD45 only |  |
| 9 | 49 | 7.5 | CD45 only |  |
| 10 | 18 | 7.5 | CD45 only |  |
| 11 | 18 | 7.5 | CD45 only |  |
| 12 | 67 | 7.5 | CD45 only |  |
| 13 | 62 | 7.5 | CD45 only |  |
| 14 | 8 | 7.5 | CD45 only |  |
| 15 | 1 | 7.5 | CD45 only |  |
| 16 | 3 | 7.5 | CD45 only |  |
| 17 | 4 | 15 | CD45 only |  |
| 18 | 2 | 15 | CD45 only |  |
| 19 | 13 | 15 | CD45 and anti CD36 |  |
| **20** | **3** | **15** | **CD45 and anti CD36** | **Yes (UWG01CTC)** |
| 21 | 15 | 15 | CD45 and anti CD36 |  |
| 22 | NA* | 15 | CD45 and anti CD36 |  |
| 23 | 0 | 15 | CD45 and anti CD36 |  |
| 24 | 118 | 7.5 | CD45 and anti CD36 |  |
| 25 | 26 | 15 | CD45 and anti CD36 |  |
| 26 | 5 | 7.5 | CD45 and anti CD36 |  |
| 27 | 19 | 15 | CD45 and anti CD36 |  |
| 28 | 7 | 15 | CD45 and anti CD36 |  |
| 29 | 1 | 15 | CD45 and anti CD36 |  |
| 30 | 1 | 7.5 | CD45 and anti CD36 |  |
| 31 | 6 | 15 | CD45 and anti CD36 |  |
| 32 | 5 | 15 | CD45 and anti CD36 |  |
| 33 | 16 | 15 | CD45 and anti CD36 |  |
| 34 | 15 | 15 | CD45 and anti CD36 |  |
| 35 | 14 | 15 | CD45 and anti CD36 |  |
| 36 | 1 | 15 | CD45 and anti CD36 |  |
| 37 | 2 | 15 | CD45 and anti CD36 |  |
| 38 | 137 | 15 | CD45 and anti CD36 |  |
| 39 | 77 | 15 | CD45 and anti CD36 |  |
| 40 | 12 | 15 | CD45 and anti CD36 |  |
| **41** | **109** | **15** | **CD45 and anti CD36** | **Yes (UWG02CTC)** |

*No CTC enumeration due to specimen clotting

**Supplementary Table 2**: STR analyses

|  | **Sample Name** | | | |
| --- | --- | --- | --- | --- |
|  | **Patient 20** | **Patient 41** | | |
| **Loci/Maker** | **UWG01CTC** | **FFPE** | **UWG02CTC** | **UWG02ASC** |
| D5S818 | 10 | 12 | 12 | 12 |
| D13S317 | 9,12 | 12 | 12 | 12 |
| D7S820 | 11,12 | 9,11,12 | 11,12 | 11,12 |
| D16S539 | 10,11 | 11,12,13 | 11 | 11 |
| vWA | 17 | 14,16 | 16 | 16 |
| TH01 | 6 | 6,7 | 6,7 | 6,7 |
| Amel | X,Y | X,Y | X,Y | X,Y |
| TPOX | 8 | 8,10 | 8 | 8 |
| CSF1PO | 12 | 12 | 10,12 | 10,12 |
| D8S1179 | 13,15 | 11,13 | 11,13 | 11,13 |
| D21S11 | 32.2 | 30,31 | 30,31 | 30,31 |
| D3S1358 | 15,16,18 | 15,16 | 15,16 | 15,16 |
| D2S1338 | 19 | 19 | 19 | 19 |
| D19S433 | 14,15 | 13,15 | 13,15 | 13,15 |
| D18S51 | 17 | 14,17 | 14 | 14 |
| FGA | 24 | 20,21 | 22,23 | 22,23 |
| Penta E | 12 | 12,16 | 7 | 7 |
| Penta D | 9 | 9,14 | 9,14 | 9,14 |

**Supplementary Table 3**: Short nucleotide variants and copy number variant analysis of DNA using the Oncomine Comprehensive Assay. Similar SNVs/CNVs were present in respective CTC line-derived mouse xenografts (data not shown).

A. UWG02CTC

| Genes | Functional effect | Coding | Amino Acid Change |
| --- | --- | --- | --- |
| *CTNNB1* | Gain-of-function | c.98C>T | p.Ser33Phe |
| *PIK3CA* | Gain-of-function | c.1252G>A | p.Glu418Lys |
| *PIK3CA* | Gain-of-function | c.1624G>C | p.Glu542Gln |
| *MET* | Gain-of-function | c.3029C>T | p.Thr1010Ile |
| *CREBBP* | Loss-of-function | c.4336C>T | p.Arg1446Cys |
| *SMAD4* | Loss-of-function | c.1609G>C | p.Asp537His |
| *GNAS* | Gain-of-function | c.602G>A | p.Arg201His |

B. UWG01CTC

| Gene alteration | Functional effect | Hg19 coordinates | Cytoband | Length | Copy Number |
| --- | --- | --- | --- | --- | --- |
| ALK  Amplification (CNV) | Gain-of-function | chr19:30303882-30314693 | 19q12 | 26.648kb | 4.8 |
| *CCNE1*  Amplification (CNV) | Gain-of-function | \| chr2: 29419631-29446279 \| \| --- \| | 2p23.2 | 10.811kb | 7.57 |

**Supplementary Table 4:** Drug IC_50_ values for UWG0^1^2ASC and UWG02CTC (from P1 – 16^1^; all hypoxic conditions)

|  | UWG02ASC | UWG02CTC |
| --- | --- | --- |
| **Drugs** | **IC_50_ (µM)^2^** | |
| Carboplatin | 9.52 ± 1.26 (n = 3) | 30.5 ± 8.5 (n = 4) |
| 5-FU/DF | 0.98 ± 0.25 (n = 4) | 1.51 ± 0.12 (n = 5) |
| Doxorubicin | 0.011 ± 0.005 (n = 3) | 0.02 ± 0.009 (n = 3) |
| Etoposide | 0.022 ± 0.007 (n = 3) | 0.036 ± 0.021 (n = 3) |
| Oxaliplatin | 0.15 ± 0.01 (n = 3) | 0.182 ± 0.052 (n = 6) |
| Paclitaxel | 0.013 ± 0.007 (n = 4) | 0.008 ± 0.003 (n = 3) |

^1^From establishment of pure culture. ^2^Mean ± SEM (of n separate experiments as shown).

**Supplementary Table 5:** Drug combination experimental values (from representative experiments)

A. UWG01CTC

|  | | | | DRI | |
| --- | --- | --- | --- | --- | --- |
| **Carboplatin (µM)** | **Etoposide**  **(µM)** | **Fa** | **CI** | **Carboplatin** | **Etopside** |
| 5 | 0.3125 | 0.374 | 0.779 | 3.119 | 2.183 |
| 10 | 0.625 | 0.516 | 0.834 | 2.909 | 2.038 |
| 20 | 1.25 | 0.656 | 0.892 | 2.720 | 1.908 |
| 40 | 2.5 | 0.792 | 0.847 | 2.863 | 2.010 |
| 80 | 5 | 0.924 | 0.485 | 4.995 | 3.514 |

DRI = Dose reduction Index, CI = combination index, Fa = effect,

B. UWG02CTC

| 5-FU (µM) | Oxaliplatin (µM) | Fa | CI |
| --- | --- | --- | --- |
| 0.1875 | 0.0625 | 0.435 | 1.219 |
| 0.375 | 0.125 | 0.747 | 0.945 |
| 0.75 | 0.25 | 0.907 | 0.851 |
| 1.5 | 0.5 | 0.962 | 0.925 |

**Supplementary Table 6**: CTC culture media

|  | **Supplier (Cat No.)** | **Concentration** |
| --- | --- | --- |
| **Serum Free Media** | | |
| Advanced DMEM/F12 | Sigma Aldrich (12491015) |  |
| N2 Supplement | Life Technologies Australia Pty Ltd (17502001) | 1x |
| Epidermal Growth Factor | Life Technologies Australia Pty Ltd (PHG0311) | 20ng/ml |
| Fibroblast Growth Factor | Life Technologies Australia Pty Ltd (PHG0266) | 10ng/ml |
| Penicillin Streptomycin Solution | Sigma-Aldrich (P4333) | 1X |
| L-glutamine | Sigma-Aldrich (G6392) | 2mM |
| **10% Serum Media** |  |  |
| Advanced DMEM/F12 | Sigma Aldrich (12491015) |  |
| Foetal Bovine Serum | Bovogen | 10% |
| Epidermal Growth Factor | Life Technologies Australia Pty Ltd | 20ng/ml |

**Supplementary Table 7**: Antibodies used in immunohistochemical analyses on cell lines

| **Antibody** | **Supplier** | **Dilution** | **Retrieval Time (min)** | **Product Code** |
| --- | --- | --- | --- | --- |
| CAM5.2 | Leica | 1:100 | 20 | NCL-L-5D3 |
| CK-7 | Leica | 1:50 | 20 | NCL-L-CK7-560 |
| CK-20 | Leica | 1:100 | 30 | NCL-L-CK20-561 |
| E-Cadherin | Leica | 1:25 | 20 | NCL-L-E-CAD |
| HER-2 | Leica | 1:100 | 20 | NCL-L-CB-256 |
| Ki67^#^ | Dako | 1:40 | 20 | Clone MIB-1 |
| Leukocyte Common Antibody (CD45) | Dako | 1:500 | 20 | CLONES 2B11 + PD7/26 |
| Vimentin | Dako | 1:500 | 20 | CLONE VIM 3B4 |
| CDX-2 | Dako | 1:50 | 30 | CLONE DAK-CDX2 |
| CD56 | Leica | 1:1 | 20 | NCL-L-CD56-504 |
| CHROM | Leica | 1:200 | 20 | NCL-CHROM-430 |
| Synapatophysin | Leica | 1:100 | 20 | NCL-L-Synap-299 |
| Urokinase Plasminogen Activator Receptor | Dako | 1:100 | 60 | Clone R4 |
| N-Cadherin^#^ | Sigma-Aldrich | 1:100 | 60 | Clone 6A9.2 |

^#^ Species reactivity not restricted to human
